# The American Medical Association and the Medical Schools

**Published:** 1858-01

**Authors:** 


*The American Medical Association and the Medical Schools.*

If we are to take the action of the late session of the American Medical Association as an indication of the feeling of the profession at large, then the evil which has so long been imminent is already upon us—that, namely, of a collision between the association and the schools, of such a nature as shall admit of no compromise, and can have no termination but the destruction of one or the other, or else a third denouement scarcely less to be deprecated, and in reality more probable, namely, an irreconcilable schism between the parties. We have now had time, since the close of the sittings, to consider, in all their bearings, present and prospective, the various measures presented to the, Society, and lying over for action at the next session, and we confess that we have arisen from the study with undiminished anxiety for the result. We have also had the opportunity of perusing what our contemporaries of the medical press have thought and said upon the subject, so far as they have taken cognizance of it. Here we have been chiefly struck with the cautious reserve which our better class of periodicals have shown in taking up the subject. We have too high an opinion of our contemporaries to attribute this to indifference, and must assign it to a creditable apprehension of increasing existing animosities, and adding confusion to existing complications, that they have preferred saying nothing to the chance of saying something which might aggravate our present difficulties.

Yielding high respect to the motives which dictate this course, we cannot feel ourselves justified in following the example. When a railway train is hurrying to a collision, apparently inevitable, it may be well for private passengers to shut their eyes and see nothing until the catastrophe occurs, but those whose duty it is to manage the train have no right to look upon the disaster as inevitable till it has occurred; they are bound in duty to keep their attention awake to the occurrence of any expedients which even to the last moment may promise to avert or even diminish the danger—and having undertaken a position, however humble, as a contributor to the periodic literature of the medical profession, we do not think we should be standing up to our responsibilities were we to decline the consideration of the momentous topics now presented to us. When it becomes necessary, as it will next May, to come to a decisive action upon these subjects, we can imagine no state of things so sure to end in disaster as that under which no one has formed a definite conception what is to be done, or what are the interests at stake, or what is the probable result of this or that line of policy, or how far each man's responsibilities extend in reference to the final event. We have deemed it our duty then, to devote our most serious attention to these matters, and, such as they are, shall present the results to our readers, only premising that we have arrived at our conclusions under a deep sense of responsibility, not disguising to ourselves the error of the one party or the other, not especially believing that any success of the one party which should humiliate or degrade the other could possibly be a matter for congratulation, or forgetting, because we hold a chair in a medicai college, that our interests as a *physician* far transcend those which affect us as a *professor*. This, indeed, is, as we look upon it, the great error of most of the parties who have undertaken the matter; they seem to have assumed that there was a necessary antagonism between the members of colleges and those members of the profession at large who desired to establish a high standard of professional qualification—forgetting that every professor is a physician, and that whatever affects the profession for good or evil affects him nearly and intimately as if he were not occupied in teaching as well as in practice.

To proceed, then—the portion of the society's proceedings which we propose to discuss are the following:

Firstly, the resolutions of Dr. Currie, which run as follows:

WHEREAS, The subject of medical education has been committed at each annual session to standing committees, and various suggestions have been proposed, which the association has adopted, and recommended to private instructors and to the medical colleges,

*Resolved,* That a committee of five be appointed by the committee on nominations, as a special committee, to be composed of members who are in no respect connected with any medical school, to *devise a system of medical instruction,* to be presented for the consideration of this association at its annual session in 1858.

*Resolved,* That the proposed system shall set forth a uniform basis, upon which our medical institutions shall be organized, as well as have reference to the best mode of securing the preparatory medical instruction to the student, and that consequently the legitimate subjects to be embraced in said system will include primary medical schools, the number of professors in medical colleges, the length and number of terms during the year, the requisite qualifications for graduation, and other such subjects of a general character as to give uniformity to our system, and preserve harmony and friendly intercourse in the ranks of the profession.

*Resolved,* That upon the adoption of the proposed system by the association, all institutions which may conform to it shall be entitled to representation at the annual sessions of this association, and none others.

These resolutions were carried against a set previously offered by Dr. Boring, to the following effect:

*Resolved*, That this association has not the power to control the subject of medical education.

*Resolved*, That the great objects of this association are the advancement of medical science and the promotion of harmony in the profession.

*Resolved*, That the attempt upon the part of this body to regulate medical education having most signally failed in its object, and already introduced elements of discord, any further interference with this subject would not only be useless, but calculated to disturb and distract the deliberations of this association.

It may be added that the committee appointed upon Dr. Currie's resolution consisted of Drs. James R. Wood, of New York; Geo. R. Grant, of Memphis, Tenn.; John Watson, of New York; C. B. Nottingham, of Macon, Ga.; Rene La Roche, of Philadelphia.

And Dr. Lindesley's proposed amendments to the constitution:

"In Art. II, omit the words 'medical colleges,' and also the words 'the faculty of every regularly constituted medical college or chartered school of medicine, shall have the privilege of sending two delegates.'"

The amendment lies over until the next meeting of the association, under a rule of the organization.

The operation of Dr. Lindsley's alteration of the constitution would be to exclude from the meetings of the association all delegates from medical colleges.

It would appear, then, that the first object in the estimation of reformers, is to deprive the schools of all voice in the proceedings, and then to devise a scheme, without their concurrence, without their having an opportunity to make a suggestion, and propose it to the schools, which are, we suppose, either to accept it unconditionally, or—or what?—quietly give up the ghost, or proceed on their work as they have done, leaving the association to do the same. Here are three different ways of dealing with the question, not one of which, we confess, is at all satisfactory to ourselves.

And first let us discuss the question, whether it is just and wise to expect the schools to accept, unconditionally, a plan, from the concoction of which they have been carefully excluded—whether they are likely to do so—whether they ought to do so. This is a question involving several considerations, foremost among which comes the constitutional point, is it right that the schools, who are numerically weaker than the medical societies, should stand on equal terms with them in regard to representation in the society? This, we understand, was made a very prominent topic of declamation by those who think the schools should be shorn of their influence; nor have we heard of any important arguments on the other side; yet we are far from thinking that the wisdom of those who originally established the principles of representation in that body is incapable of defense. In no representative body, consisting of but one chamber, but representing a diversity of interests, is or ought the representation to be based merely upon numerical considerations. In the political institutions of our country it is obvious that, if we had but one legislative chamber, and that the house of representatives, with its numerical basis, the less populous states of the south, with their peculiar commercial and social interests, would be completely at the mercy of the more densely peopled eastern and northern states. This evil is balanced by the provision of a separate chamber (the Senate), in which all the States, populous or not, are equally represented; and thus, in a measure, is the probability obviated, of a numerical majority so legislating as to act oppressively upon the interests of states not able numerically to contest the points with them. Now, in our national medical association we have but one chamber; and if there be (as there undoubtedly is) in the profession a diversity of interests, it is both just and wise that the rights of any one of the parties to it should be secured from infraction, by such an adjustment of the representative basis as shall enable it to be heard with effect.

Now, it needs very little argument to show that the teaching portion of the profession stands exactly in this position. The national association claims (and never so peremptorily as now) to dispose of everything relating to medical instruction, and that absolutely and without limit. If the language of some of the present advocates of change is to be taken as the true exposition of its powers, it can legislate all existing schools out of existence, and establish a new system of education in their place. If, then, the interests and very existence of the medical schools is at the mercy of the association, is it not a most reasonable provision that they should have their representatives in that body to vindicate their peculiar rights and interests? Is it right that a body of men like the faculties o the medical schools of this country, possessing, we are bold to say, an aggregate of talent and character at least equal to that of any body of men in the nation who could be similarly associated in interest, should have their rights and interests, and very existence, at the mercy of a council board in which they have no voice? We have, for argument's sake admitted that the numerical assumption is correct, that the delegates from medical societies *do* represent a vastly greater number of physicians than those from the schools; but we very much doubt whether, after all, this, is practically the case—whether it is anything more than one of those figments upon which so many political arrangements are grounded. We have become acquainted with the details of more than one society, formed for the express purpose of sending delegates to the meeting of the general association, and we leave it to our readers, who are similarly informed, whether what we are now going to describe is not a correct delineation of such proceedings in a great many instances, whether it may not, indeed, (to borrow a word from pathology,) be received as a typical case, exemplifying a class rather than an individual instance. In a county or city, as the case may be, some few months before the meeting of the general Association, some physician, (or physicians,) fond of individual prominence, is suddenly struck with the necessity of having the profession in the county or city aforesaid represented in the national conference. He holds conversations, say, with five or six other physicians, who are brought to a similar conviction, and the half dozen of them go about and talk their best, or use whatever other means of influence they may possess with their brethren to induce a sufficient number to meet together and organize a new society or revive an old one, and then, after electing officers, etc., the original concoctor or concoctors are appointed delegates—very deservedly and proper—and the society goes to sleep again, till another season for appointing delegates evokes its torpid vitality. Now, who do the delegates in question represent—we mean practically, apart from all legal factions? Not the unheeding members who have been talked, and begged, and urged into coming there for the purpose of an organization, and who care no more about the society than about parliamentary organizations in the moon. But at the utmost the few who exerted themselves to get up the society, if even those few, care enough about it to have it kept up after the object is subserved. We are certain of this, to come down from generality to particulars, that in Memphis, if if had not been for the active exertions of members of the Medical College, the society which sent a delegate to represent the profession of that city in the recent national association would have been a very small affair indeed—would have consisted nearly exclusively of the personal friends of the excellent physician who was ultimately appointed, and who so creditably sustained the character of Memphis in the national councils. And since the session of that body, the two or three who have been gathered together at the times for the regular meetings of the society, in the vain hope of a quorum, have consisted exclusively of members of the college.

Considering all these circumstances, then, we doubt whether, even numerically, the constituents of the members sent by societies are practically more numerous than those sent by the colleges, especially if from the constituency of the former be deducted those physicians who are also professors. In the Memphis Medical Society, with not more than one or two exceptions, the members of the college have been the only men who have taken an active and permanent interest in keeping it up, and if it has been objected to, that it has been a mere creature of the college, the only color to the assertion has been that the college faculty adhered to it and kept it up after every one else had deserted it. Let us here boldly assert our belief that the preponderance of college interests in the American Association, if real, has had a similar cause. Assuredly the number of societies which exist, or might be formed if the profession took sufficient interest in the matter, is vastly greater than that of the medical colleges, so that there is every facility for outnumbering the college delegates by those from societies representing the profession at large—and if this has not been done, why is it so? Not surely from any inherent defects in the constitution of the society, but from the greater interest which the members of the colleges take in the public concerns of the profession than do the physicians at large.

Alas, if the purposes so plainly and eagerly expressed at the last meeting of the association be really carried out, and the delegates from colleges be excluded from its councils, we fear it would become painfully apparent how little claim that body had to be considered as representing the medical profession of the United States. If the schism toward which recent events so ominously point should really take place (which may God avert), we know not what might be the effects on the colleges, but the association, we feel sure, would become deplorably insignificant. We are farfrom looking upon this as a desirable consummation; we desire for the association a long and useful existence—we wish that it more really and practically represented the whole profession than it does; but we think it absurd to suppose that its position and influence would be stronger without the concurrence of colleges.

But to descend from general constitutional considerations to the conjuncture now before us, we would put the question; is the principal problem now at issue, the improvement of the system of medical education, likely to be better resolved with or without the intervention of the medical schools in the deliberations relating to it? We are not here calling in question the attainments of any of the eminent men placed upon the committee for this purpose. As a learned cultivator of medical literature and as a skillful and practical physician and surgeon, we do not suppose that two abler men than Rene La Roche and Jas. R. Wood could have been selected out of the whole profession, either within or without the schools, and we know our fellow-citizen, Dr. G. Grant, for a public spirited and well informed physician, who loves his profession. It is true that the exclusion of Dr. Currie from the committee appointed upon his own resolutions is a matter which yet remains to be accounted for; yet with all omissions it is a good committee, as good as we could wish, supposing it right that members of the medical schools should be excluded. But this provision, we are convinced, could not detract much and add nothing to the efficiency of the committee. For let us grant that these gentlemen were as competent as could be found for devising a scheme for medical education, excellent in the abstract, there is yet another set of considerations which surely could not be so completely dealt with by them as by men now actually engaged in the duties and responsibilities of medical education. We allude especially to those considerations which arise out of the local circumstances of the field in which this educational scheme has to be put in action. How are these gentlemen, writing their books and practicing the duties of their profession in Philadelphia, New York, etc., to form a practical estimate of the feasibility of this or that scheme, and its adaptation to peculiar circumstances, and its competency to deal with peculiar difficulties, here in the valley of the Mississippi? for we apprehend that adaptation to peculiar circumstances, and practicability under peculiar difficulties, are as essential qualifications of the forthcoming scheme, as the abstract excellence of its standard attainment and method of instruction. We have already, in a former number of this journal, endeavored to show that the standard of attainment for the average medical practitioners in a given community, must grow out of its peculiar circumstances. We there pointed out that an arbitrary standard, one derived from a different condition of society, could never be successfully transplanted to an uncongenial soil; but, not to be at the labor of repeating the same observations in different language, we quote from the article in question:*

"Let us consider what is the standard in England: The candidate must there, first of all, by examination, show himself to be in possession of a thorough liberal education, and under that term is included all that *we* mean by a complete university course; all this, I say, is to be determined by an examination before the medical course is commenced upon; but an academical degree in one of these public universities is allowed to supercede this examination. Such a degree costs those who receive it

*Memphis Medical Recorder, May, 1856, p. 355.

three and a half years' study and about $4,000 or $5,000 to obtain, after the school education, which lasts till about the age of eighteen, is complete. Supposing all this to be gone through with, the proper medical education commences, which, including hospital attendance, etc., cannot occupy less than five years, the expenditure already specified being at least doubled during the time. Now, suppose that we were to adopt this rule here—suppose it were to be adopted throughout the United States, does any one suppose that there would be a single graduated M. D. outside of the great eastern cities, or inside of them, anything like sufficient for the mere city practice? Why, the money expended in it alone would, in this country, be a small fortune, and if used judiciously for the number of years I have specified, in any one of the boundless fields open in this great country for the investment of capital, would, by the time your Englishman is ready for his diploma, have amounted to a very large fortune, as fortunes go here. This must be enough, then, to make it obvious that the amount of time and capital which young men have to possess in order to attain to other respectable modes of acquiring a position and making a subsistence in the world, is the measure of the materials which we possess for making doctors of. If we wait for anything more than this, we might as well wait for the Mississippi to flow past before we step over into Arkansas. We must do the utmost we can with the materials furnished to us, and not spend our time in a useless sighing after what cannot be had.

Another circumstance likely to affect this question is the average amount of remuneration a physician is likely to receive for his practice when he has got his diploma; for men are not ready to spend much labor, time and money upon preparation for a profession which is likely to make an ungrateful return for the expenditure. Now the labor, time and capital which the student is willing to spend upon his apprenticeship constitute the material out of which new physicians have to be produced; and those who are without a practical knowledge of these resources, their amount and nature, are no more fitted to legislate for the emergency than would an architect be to contract for a large building, who was skilled, indeed, in the proportions of Corinthian facades and Palladian domes, but who was ignorant of the physical properties of wood and stone and iron.

Perhaps a better illustration could not be given of the fertility of trying to establish a standard of qualification unadapted to circumstances than may be drawn from the history of the profession in England. The physicians of old in that country were great sticklers for keeping the profession exclusive. This was to be done, not only by requiring a very long period of probation before conferring a degree, but by making the process very expensive, requiring exorbitant fees at various stages. This was done avowedly for the purpose of keeping the profession select. They would defend their mode of proceeding by contending that it was necessary to provide that the physician should be a *gentleman* as well as a scholar—rather a snobbish idea of a gentleman, to be sure, where the length of his purse was to establish his claim to the title. But, however, the custom was sufficiently successful to establish the regular *diplomats* as a sort of professional aristocracy who held their heads very high among the various social circles into which the middle classes were divided; but another result was that the number of persons entitled by diploma to write themselves M. D. was ridiculously small compared with the amount of work to be done; and, moreover, as will happen under such circumstances, their fees were so high that it was utterly out of the power of the people at large to employ them. The inevitable consequence of all this was that some one or other not stamped witn a diploma should step in and do the work. This was, in fact, done by the apothecaries of the time. These men were, by law, only authorized to dispense the prescriptions of the physicians; but, from first selling a few of the simpler medicines, with directions for their use, they gradually went on to occasionally prescribe for minor diseases at the counter, to draw teeth and perform other minor operations in surgery; then they would visit patients, and finally got employed in all the cases that required the aid of physicians and surgeons, by those who could not afford to employ a regular M. D. Within the present writer's recollection it was quite common with families in the country to employ a physician for their own ailments, while the apothecary was called in when the servants got sick. Well, at first the prescribing apothecaries were bitterly opposed by the physicians, who, in truth, were not without fair pretext for doing so, as many of them were excessively incompetent; but, gradually, as population increased, the practicing apothecaries became more numerous, the titled physicians fewer, the apothecaries better educated, the physicians less manifestly their superiors; and, especially, as there came to be a large field of practice which the physician could not undertake, the apothecary came to be in some sort recognized by the physicians; they would even meet in consultation —if consultation it could be called—where the apothecary was barely allowed to call his soul his own; the truth is, that they only came together at all when a case in the hands of an apothecary became serious and the family alarmed, and some eminent physician from a distance was called in, under which circumstances the apothecary was retained, but was expected simply to carry out the prescriptions of the consulting physician, but in no case to prescribe himself, except in some emergency arising in his absence. Moreover, the status of the apothecary came to be partially recognized by the state. In some instances he was employed by the state, as in attendance on jails, almshouses, &c.—especially where the salary was beneath the notice of the more lordly physician. But this state of things was getting unsatisfactory to all parties. Not only was the apothecary, (though now a well educated gentleman in many instances) liable to these mortifications, but it was illegal for him to recover a bill for medical treatment except in the form of charging for medicine, and thus is supposed, among other results, to have arisen the excessive medication which characterized English medical practice fifty years ago. Another result was that as he practiced rather by the connivance of the law than in accordance with its provisions, no law could relate the qualifications required of him before he could practice. So that, though many men practicing as apothecaries possessed very high qualifications, yet there were many more who were utterly defective in their medical education. In consequence, a succession of acts of Parliment were passed at different times endeavoring to define the nondescript position of the practicing apothecary, and finally he was established in a definite legal position, with his name changed, under the title of a general practitioner. This title was given to indicate that the possessor of it practiced both medicine and surgery, it being considered *infar dig.* either for the physician to practice surgery or the surgeon to prescribe otherwise than for a surgical case—a feeling which is still kept up so far that it is provided that the Colleges of Physicians and Surgeons shall be presided over by a pure physician and a pure surgeon, respectively; i. e., by gentlemen neither of whom has soiled his delicate hands by stepping beyond his *specialty*.

The state of things is this, then, at present: the general practitioners, the successors of the licensed apothecaries, are to all intents the physicians of the people, including in this term the respectable middle classes as well as the poor, the physicians proper and the pure surgeons forming a kind of professional aristocracy among them, more or less recognized according to the talents and success of the individauls claiming rank as such.

Now, it seems to us, that this simple narrative is full of instruction most valuable for us: we are specially fond of pointing out the errors of older countries, we are much less willing to learn wisdom from their mistakes. But let us suggest that if we should be guilty of an error similar to that of the profession in England, there is no hope of its being retrieved with as little injury as it was in that country by legislative enactment. There, as soon as it was seen that the needs of the country could not be filled except by legalizing the practice of the apothecary, under the title of general practitioner, it was found possible to establish him not only with his rightful privileges and emoluments, but with proper checks and securities for his training and competency. But, can this be expected here? Suppose that by adopting an impracticable standard of qualification for physicians, we should throw the whole of the growing population of these western states into the hands of untrained, unqualified, and unrecognized practitioners—as would undoubtedly be the case—what then could our legislatures be reasonably called upon to do to remedy the evils which professional want of foresight had brought about? What are we to infer, from their previous actions in regard to our profession, what would the spirit of their legislation be, if they consented to legislate at all? We implore our committee and other parties to the solution to put this question earnestly to themselves before they take steps, which, if once taken in the wrong direction, are obviously irretrievable. If, then, the circumstances which affect the practicability of any scheme proposed are so momentous a topic for consideration, who—we would repeat the question—who are the persons most likely to be possessed of information upon the subject? Who but those who have spent their lives in the practical efforts to elevate the standard of education, and have thus become practically acquainted with the difficulties which environ the subject? Who but the existing medical teachers, whom it is the proposition to exclude altogether from the national councils of the profession, and who have already been excluded from the treatment of this question? The only ground that has been alleged for doing so, viz: that it is contrary to the interest of existing schools to improve as far as practicable the existing plan of education, we shall not condescend to answer or to take into a moment's consideration; we will only observe that we know of no one in the profession who has advocated with so much zeal and intelligence the cause of educational reform as Drs. Wood and Gross of Philadelphia, Dr. Fenner of New Orleans, and, with, perhaps, humbler claims to attention, but certainly with a zeal no less ardent, we claim to have been now for a long time a consistent advocate of all practicable reformation, and to have exerted our best abilities in pointing out where defects existed, and how they might be remedied. Doubtless, there exist temptations in this occupation as well as in all others to make private emolument a stronger motive than the public good, and doubtless these temptations have in some instances been yielded to, but we certainly see no security for supposing that if the duty of instructing the rising generation of physicians were withdrawn from the hands which at present exercise it, a body of men more conscientious and more zealous for the well-being of the profession would be substituted.

It remains to be considered what ought to be the course pursued by the teaching members of the profession in reference to the questions to be brought before the next meeting of the national association; and, of course, no definite course can be chalked out till the aspects and probable line of action to be pursued by that body can be calculated upon. Our own expectation is, that so far as the action of the last convention was hostile to the teaching members, it will be reversed in the next. We have already shown that from the non-attendance of delegates from distant States, and the preponderance of certain local influences, the last meeting cannot in any sense be looked upon as representing the general state of feeling throughout the profession, and we shall not be surprised to see the motion of Dr. Lindsley laid on the table, while the committee appointed under Dr. Currie's resolutions will doubtless report, and its report be treated with courtesy; but, we anticipate no attempt to *enforce* its recommendations, and thus the only results of the unfortunate line of action pursued by the association would be to defer for a year longer any concerted action on educational reform. Should this be the case, we think that then the schools themselves should initiate some well concerted plans of improvement to be presented to the meeting of 1859 for its approval. On the other hand, should the action of the association be confirmatory of that of last May, then we think the schools should undertake the matter in concert with one another, but entirely apart from the association—leaving that body to carry out its own measures. This position of things we earnestly deprecate, however, as leading obviously to a formal schism between the schools and the association. We trust the conjuncture will not be presented.—*Memphis Med. Recorder.*

